# Pamidronate improves the quality of life and induces clinical remission of bone metastases in patients with thyroid cancer

**DOI:** 10.1054/bjoc.2001.1832

**Published:** 2001-06

**Authors:** G Vitale, F Fonderico, A Martignetti, M Caraglia, A Ciccarelli, V Nuzzo, A Abbruzzese, G Lupoli

**Affiliations:** Dipartimento di Endocrinologia ed Oncologia Molecolare e Clinica, Facoltà di Medicina e Chirurgia, Università degli Studi “Federico II”, Via Pansini 5, 80131, Napoli; Dipartimento di Biochimica e Biofisica, II Università di Napoli, Napoli, 80100, Italy

**Keywords:** pamidronate, thyroid cancer, bone metastases

## Abstract

Skeletal metastases from thyroid cancer are poorly responsive to medical or radioiodine treatment. Bone destruction in skeletal metastases results from osteoclast-induced bone resorption. Therefore, a new approach in the therapy of bone metastases consists in using aminobisphosphonates, such as pamidronate, which are potent inhibitors of osteoclastic activity. In the present study, 10 thyroid cancer patients with painful osteolytic bone metastases were administered pamidronate (90 mg, as a 2 hour intravenous infusion) monthly for 12 consecutive cycles. Bone pain, quality of life, performance status, analgesic consumption and disease staging were evaluated before and during the trial. The patients who had been administered pamidronate showed a significant decrease in bone pain (*P* = 0.0052). Performance status improved nearly significantly (*P* = 0.051), while the quality of life showed a remarkable amelioration. However, no significant decrease in analgesic consumption was recorded. Partial radiographic response of bone lesions was observed in 2/10 patients. The side effects of pamidronate were mild and transient. In conclusion, monthly infusion of pamidronate is a well-tolerated treatment that induces significant relief from bone pain and improves the quality of life of thyroid cancer patients with symptomatic and osteolytic bone metastases. © 2001 Cancer Research Campaign http://www.bjcancer.com


*British Journal of Cancer* (2001) **84**(12), 1586–1590
© 2001 Cancer Research Campaign
doi: 10.1054/ bjoc.2001.1832, available online at http://www.idealibrary.com on IDEAL®

http://www.bjcancer.com

### Pamidronate improves the quality of life and induces clinical remission of bone metastases in patients with thyroid cancer

**G Vitale[1], F Fonderico[1], A Martignetti[1], M Caraglia[2], A Ciccarelli[1], V Nuzzo[1], A Abbruzzese[2] and G Lupoli[1]**

[1]Dipartimento di Endocrinologia ed Oncologia Molecolare e Clinica, Facoltà di Medicina e Chirurgia, Università degli Studi “Federico II”, Via Pansini 5, 80131, Napoli; [2]Dipartimento di Biochimica e Biofisica, II Università di Napoli, 80100, Napoli, Italy

**Summary** Skeletal metastases from thyroid cancer are poorly responsive to medical or radioiodine treatment. Bone destruction in skeletal metastases results from osteoclast-induced bone resorption. Therefore, a new approach in the therapy of bone metastases consists in using aminobisphosphonates, such as pamidronate, which are potent inhibitors of osteoclastic activity. In the present study, 10 thyroid cancer patients with painful osteolytic bone metastases were administered pamidronate (90 mg, as a 2 hour intravenous infusion) monthly for 12 consecutive cycles. Bone pain, quality of life, performance status, analgesic consumption and disease staging were evaluated before and during the trial. The patients who had been administered pamidronate showed a significant decrease in bone pain ($P = 0.0052$). Performance status improved nearly significantly ($P = 0.051$), while the quality of life showed a remarkable amelioration. However, no significant decrease in analgesic consumption was recorded. Partial radiographic response of bone lesions was observed in 2/10 patients. The side effects of pamidronate were mild and transient. In conclusion, monthly infusion of pamidronate is a well-tolerated treatment that induces significant relief from bone pain and improves the quality of life of thyroid cancer patients with symptomatic and osteolytic bone metastases. © 2001 Cancer Research Campaign http://www.bjcancer.com

**Keywords**: pamidronate, thyroid cancer, bone metastases

Thyroid cancer is a relatively rare disease which is usually curable. However, in some patients, it can have an aggressive course leading even to death. In fact, thyroid cancer shows wide variations in the degree of malignancy, ranging from an almost benign to an extremely aggressive type of cancer (Gillenwater and Weber, 1997).

Thyroid cancer metastasizes most frequently to cervical lymph nodes, but also to lung and bone. The occurrence of skeletal metastases, which are often osteolytic, is associated with a high risk of death (Marcocci et al, 1989). Moreover, bone metastases can be responsible for pain, swelling, pathological fractures, neurologic deficits, impaired mobility, hypercalcaemia and worsening of the quality of life (Coleman and Rubens, 1985; Body, 1992; Schlumberger, 1998).

Although the induction of the remission of the disease and the improvement in overall survival rate remain central to cancer clinical research, it is now widely accepted that relief from symptoms and protection against skeletal complications should not be overlooked as therapeutic goals to be achieved in cancer patients with bone metastases. Therefore, the improvement in the quality of life may be as important as the increase in survival rates in these patients.

Unfortunately, the treatment of skeletal metastases represents a difficult challenge in thyroid cancer and clinical results are not encouraging. In fact, the response to the therapy of bone metastases through surgery (Schlumberger, 1998), radioiodine (Marcocci et al, 1989; Maxon and Smith, 1990; Proye et al, 1992), external irradiation (Tubiana et al, 1985), chemotherapy (Grebe and Hay, 1995; Schlumberger, 1998) and biological therapy (Lupoli et al, 1996; Vitale et al, 2000) is poor in thyroid cancer patients.

Bisphosphonates, which are potent inhibitors of osteoclastic activity, responsible for the accelerated bone resorption in osteolytic metastases, represent an alternative therapeutic approach to bone metastases due to several tumours (Cascinu et al, 1998). Pamidronate is one of the most potent aminobisphosphonate commercially available. The efficacy of pamidronate in malignant bone disease is well know (Lipton et al, 1994; Cascinu et al, 1998), although zoledronate, still under clinical investigation, seems to be more promising.

The aim of the present study was to evaluate the tolerability and activity of pamidronate disodium in relation to bone pain, quality of life, analgesic consumption and tumour mass in patients affected by thyroid cancer with painful and progressing lytic metastases of bone.

### MATERIAL AND METHODS

### Patient selection

10 patients, 7 men and 3 women, ranging in age from 35 to 76 years (mean ± SD: 57.1 ± 12 years; median: 58 years), with painful bone metastases from histologically proven thyroid carcinoma (6 follicular, 2 papillary, 2 medullary), were enrolled in this study. In

*Received 13 October 2000*
*Revised 23 February 2001*
*Accepted 19 March 2001*

*Correspondence to:* G Lupoli all patients initial treatment with surgery, radioiodine therapy, external radiotherapy, chemotherapy and/or biological therapy had been unsuccessful and they were no longer eligible for standard antineoplastic treatments at the time of admission.

The other criteria adopted for selecting patients to be included in this study were: radiographic evidence of at least one osteolytic bone lesion; absence of symptomatic heart disease; absence of significant ascites or other third-space fluid collection; no pregnancy or nursing; normal liver and kidney biochemistry (total bilirubin < 1.5 mg $dl^{-1}$, aspartate aminotransferase and alanine aminotransferase < 3 times the normal limit, prothrombin and partial thromboplastin < 1.5 times the normal limit and creatinine < 1.2 mg $dl^{-1}$); normal value of serum calcium corrected for albumin; absence of any skeletal complication in the previous 3 months (pathologic fracture, the need for irradiation or surgery of bone, spinal cord compression due to vertebral collapse); a washout period of at least 3 months before the trial from any previous treatment with antitumour agents (radioactive iodine therapy, external radiation therapy, chemotherapy and/or biological therapy), bisphosphonates or other drugs known to influence bone metabolism; an estimated life expectancy of at least 9 months.

### Treatment schedule

Patients were treated with 90 mg pamidronate disodium (Aredia, Novartis Farma S.p.A., Italy), administered as a 2-hour intravenous infusion in 500 ml of 0.9% saline. The bisphosphonate was administered monthly for 12 consecutive cycles of therapy.

No other anticancer treatment was allowed during the course of the study, with the exception of TSH-suppressive therapy with 1-thyroxine in differentiated thyroid carcinoma. This endocrine therapy had been started at least 2 years before this trial and remained unaltered while the therapy with pamidronate was being followed.

The study was approved by the Institutional Bioethical Committee and all patients provided written informed consent.

### Evaluation of treatment tolerability

Patients underwent clinical and biochemical examination before entry into the study and then monthly for 12 consecutive times. Each evaluation included complete physical examination, a routine biochemical profile, the assessment of side effects and the identification of any skeletal complications. All adverse events were recorded and graded according to World Health Organization (WHO) criteria (Miller et al, 1981). The evaluations of serum calcium corrected for albumin and of phosphate, magnesium, potassium, creatinine, haemoglobin and haematocrit were performed in the first 2 weeks following the treatment.

### Evaluation of symptomatic response

To assess symptomatic response, patients were asked to record at baseline and quarterly the average intensity of pain during the previous week by using a 100 mm visual analogue scale (VAS), where 0 stood for no pain and 100 for extremely severe pain (Huskisson scale). At the same time, patients completed the Functional Assessment of Cancer Therapy-General questionnaire (FACT-G, version 4). FACT-G is a multidimensional questionnaire developed and validated in cancer patients to evaluate the changes in the 4 main domains of the quality of life: physical well-being (7 items), social/family well-being (7 items), emotional well-being (6 items) and functional well-being (7 items). Patients scored each item on a 5-point ordinal scale range ranging from 0 to 4 (0 = not at all; 1 = a little bit; 2 = somewhat; 3 = quite a bit; 4 = very much) during the previous 7 days. The FACT-G questionnaire is available in 24 different languages and is widely accepted in the Italian language (Cella et al, 1993). At each time point, scores for Eastern Cooperative Oncology Group (ECOG) performance status according to WHO scale (Beahrs et al, 1988) and analgesic consumption were recorded. We also calculated the Trial Outcome Index (TOI), by adding the functional to the physical domain score; and a pain score was obtained by adding TOI to 100-VAS. Analgesic consumption was expressed through narcotic score. This parameter was obtained multiplying the type of pain relief medication administered (0 = none; 1 = analgesic; 2 = mild narcotic; 3 = strong narcotic) by the frequency of administration (0 = none; 1 = less than daily; 2 = once a day; 3 = more frequently than once a day) (Tong et al, 1982).

### Evaluation of tumour mass response

The presence of bone metastases was identified by radionuclide bone scan and assessed by plain radiography, computerized tomography scan or magnetic resonance imaging when necessary. In case of skeletal pain in a specific site without any evidence of disease through radionuclide bone scan, a plain radiograph of the painful area was made. These diagnostic procedures were performed before starting the study and during the therapy with pamidronate at 6 and 12 months intervals. The response of bone lesions was assessed blindly by a radiologist according to the following International Union Against Cancer (UICC) guidelines for responses in breast cancer (Hayward et al, 1977).

Complete response (CR): disappearance of all known bone diseases, including calcification of lytic bone metastases.

Partial response (PR): 50% or greater decrease in measurable bone lesions or an objective improvement in evaluable, but non-measurable bone lesions. It was not necessary for every bone lesion to have regressed, but no bone lesion should have progressed.

No change (NC): bone lesions unchanged (< 50% decrease or < 25% increase in the size of measurable lesions).

Progressive disease mixed (PDM): some bone lesions regress while others progress or new bone lesions appear.

Progressive disease failure (PDF): progression of some or all bone lesions and/or the appearance of new bone lesions. No bone lesion regresses.

Disease staging of extra-skeletal metastases was performed before the beginning of the treatment and then every 6 months throughout the treatment by chest X-ray, neck and abdominal ultrasound, total-body computerized tomography and/or magnetic resonance and response according to the WHO criteria (World Health Organization, 1979).

### Statistical analysis

Analysis of variance with repeated measures (ANOVA) was used to compare the scores of the following parameters during the therapy: VAS, FACT-G, TOI, TOI + (100-VAS), performance status and narcotic score. The most recent values were compared to baseline using the *t*-test for paired data.

© 2001 Cancer Research Campaign 

**Table 1** Characteristics of patients, tumour mass response of skeletal metastases to pamidronate and toxicity of treatment

| Patient | Sex/Age (yr) | Histology | Sites of skeletal metastases | Sites of extra-skeletal metastases | Tumour response | Side effect (WHO grade) |
|---|---|---|---|---|---|---|
| 1 | F/35 | Follicular | Spine, femur | — | PR | Fever (I) |
| 2 | F/76 | Papillary | Spine | — | NC | Nausea (I) |
| 3 | F/48 | Follicular | Spine, femur, tibia | Liver | PR | ↑ skeletal pain, ↓ calcium |
| 4 | M/60 | Follicular | Pelvis | — | NC | — |
| 5 | F/46 | Follicular | Spine, pelvis | — | NC | ↑ skeletal pain |
| 6 | M/56 | Papillary | Ribs | — | NC | — |
| 7 | F/54 | Medullary | Spine, sternum | Lung | PDM | Fever (II), myalgia (I) |
| 8 | F/66 | Medullary | Spine, sternum, clavicle | Mediastinum | PDF | Fever (I), ↓ potassium |
| 9 | F/69 | Follicular | Pelvis | — | PDF | — |
| 10 | M/61 | Follicular | Spine, ribs | — | NC | Nausea (I) |

PR = partial response; NC = no change; PDM = progressive disease mixed; PDF = progressive disease failure.

All statistical analyses were performed with BMDP statistical package (BMDP statistical software, Los Angeles, CA, USA).

### RESULTS

### Patient characteristics and treatment tolerability

Characteristics of patients with thyroid cancer and skeletal metastases are summarized in Table 1. Nine out of 10 patients completed 12 cycles of treatment with pamidronate. One patient (patient 8) discontinued the therapy after 7 cycles because of the progression of the disease and skeletal complications.

The treatment was well tolerated and minor adverse events were usually recorded only during the first cycles (Table 1). Three out of 10 patients had a transient increase in body temperature (WHO grades I–II). Myalgia (WHO grade I) and a mild increase in skeletal pain were observed in 1 and 2 patients, respectively. Nausea (WHO grade I) was reported in 2 patients. All side effects were transient, usually lasting 6–48 hours after infusion, and did not necessitate treatment discontinuation. During the treatment, plasma calcium and potassium decreased slightly in patient 3 and patient 8, respectively. No significant change was observed in haematologic, hepatic and renal parameters. Therefore, pamidronate was safe and did not induce significant side effects.

### Symptomatic response

Table 2 reports the variations in pain, quality of life and narcotic score, expressed as mean ± SD, and statistical analysis during the treatment with pamidronate. The changes in VAS scores, assessed by analysis of variance, were statistically significant ($P$ = 0.0052) and the percentage decrease in mean VAS values from baseline (Figure 1) was maximal after 3 months of therapy (–31.35%) and minimal after 12 months (–18.92%). We also measured the quality of life through the questionnaire FACT-G, that was well accepted by the patients. On average, it required 5–10 minutes to be completed and , in most cases, could be filled out by patients themselves with little or no assistance. We observed a statistically significant improvement in the quality of life as measured by FACT-G ($P$ = 0.0059) and TOI ($P$ = 0.0115), suggesting a clinically relevant impact of pamidronate therapy in patients with bone metastases from thyroid cancer. The maximal percentage increase in mean FACT-G values from baseline was +16.91% after 6 months of therapy (Figure 1). When the TOI was added to the reciprocal value of VAS score, results were also statistically signficant ($P$ = 0.0044). Matched t-test between baseline and one year value was also significant for VAS, FACT-G and TOI + (100-VAS) scores ($P$ = 0.0157, $P$ = 0.015 and $P$ = 0.0303, respectively) and this test was at the statistical significance limit for TOI ($P$ = 0.05), indicating that the benefit derived from the treatment was still present after a long-term treatment.

During the trial, a decrease in narcotic score was also observed, but this change was not statistically significant ($P$ = 0.088) when using analysis of variance. On the contrary, performance status, assessed according to ECOG scale, improved at the statistical significance limit (P = 0.051).

### Tumour mass response

Partial response of bone lesions was evidenced in 2 out of 10 patients after 6 and 12 months of pamidronate therapy (patients 1 and 3). In these 2 cases a decrease greater than 50% in vertebral and femoral lesions was observed (Table 1). In addition, 5 patients achieved stabilization of bone lesions during the trial (patients 2, 4, 5, 6 and 10). Progression of bone lesions developed in 1 patient after 6 months (patient 8) and in 2 patients after 12 months (patients 7 and 9).

**Table 2** Variations (mean ± SD) in pain, quality of life and narcotic score during the therapy with pamidronate

| | Baseline | 3 months | 6 months | 9 months | 12 months | *P* |
|---|---|---|---|---|---|---|
| VAS | 53.9 ± 17.4 | 37 ± 20.7 | 38.5 ± 18.5 | 40.9 ± 15.2 | 43.7 ± 13.1 | 0.0052 |
| FACT-G | 55 ± 16.6 | 62.1 ± 17.4 | 64.3 ± 13.7 | 63 ± 13.9 | 64 ± 12.6 | 0.0059 |
| TOI | 27.2 ± 11.3 | 30.7 ± 12.8 | 32.6 ± 10.7 | 31.6 ± 10.4 | 31.1 ± 9.94 | 0.0115 |
| TOI + (100-VAS) | 73.3 ± 26 | 94.7 ± 31.6 | 94.1 ± 27.4 | 94.1 ± 22.9 | 88.5 ± 19.2 | 0.0044 |
| Narcotic score | 4.1 ± 2.5 | 3 ± 2.2 | 2.6 ± 1.9 | 3.2 ± 2.4 | 3.4 ± 2.4 | 0.088 |

VAS = visual analogue scale; FACT-G = functional assessment of cancer therapy general; TOI = trial outcome index.

 *© 2001 Cancer Research Campaign*

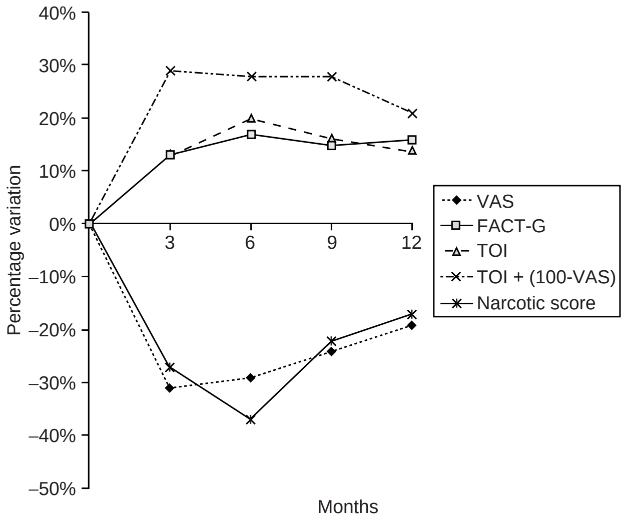

**Figure 1** Percentage variations from baseline of mean values for VAS, FACT-G, TOI, TOI + (100-VAS) and narcotic score during the trial. VAS (visual analogue scale), FACT-G (functional assessment of cancer therapy general), TOI (trial outcome index)

While evaluating extra-skeletal metastases, a stabilization in hepatic lesions in patient 3 during the therapy was observed; progression of pulmonary and mediastinal metastases occurred in patients 7 and 8, respectively.

### DISCUSSION

Aminoderivatives, such as pamidronate, are particularly potent bisphosphonates, that act through the induction of osteoclast apoptosis, probably due to the inhibition of cholesterol biosynthesis and, consequently, to the inhibition of the isoprenylation of proteins required for the osteoclast survival (Benford et al, 1999; Van Beek et al, 1999). Aminobisphosphonates are commonly administered to patients with osteolytic bone metastases caused by several neoplasms. These agents decrease the intensity of pain and the incidence of fractures thus improving mobility. Moreover, several investigators have reported that X-ray showed sclerosis of lytic bone metastases in patients treated with bisphosphonates (Coleman et al, 1988; Morton et al, 1988; Lipton et al, 1994; Cascinu et al, 1998).

These data provided the rationale for our observational study to determine the effects of the therapy with pamidronate on a selected group of thyroid cancer patients with symptomatic and lytic skeletal metastases. In these patients the conventional therapy (surgery, radioactive iodine therapy, external irradiation, chemotherapy and/or biological therapy) was unsuccessful. In the current trial a significant decrease in bone pain was observed during the therapy with pamidronate. In fact, in comparison to baseline values, an average pain reduction of 31.35% was observed after 3 months of therapy. This pain relief is the primary goal of palliative treatment for bone metastases. We also observed a remarkable improvement in the quality of life, assessed by FACT-G, during the therapy. It must be noted that the effects of pamidronate on bone pain and quality of life continued throughout the 12 months of therapy, thus suggesting a continued effect of pamidronate in long-term treatment. Interestingly enough, the appearance of areas of sclerosis in lytic bone metastases induced a partial response in 2 out of 10 patients and, in addition, in 5 out of 10 patients the disease stabilized during therapy.

In light of previous studies, a direct antitumour effect of pamidronate cannot be excluded. In fact, pamidronate has been recently reported to have a cytostatic effect and to induce apoptosis in multiple myeloma cells. These effects cannot be solely justified by a secondary decrease in the production of the myeloma growth factor interleukin-6 (Shipman et al, 1997; Aparicio et al, 1998; Derenne et al, 1999). It could be assumed that the anti-isoprenylating activity of aminobisphosphonates is responsible for the growth inhibitory effects (Reszka et al, 1999). However, data on thyroid tumour cells are not available yet.

During this trial, no specific anticancer therapy was performed with the exception of TSH-suppressive therapy with 1-thyroxine in differentiated thyroid cancer. However, this endocrine therapy was started before the study and was not modified during the therapy with pamidronate. Hence, the symptomatic improvements and the effects observed on tumour mass can potentially be attributed to pamidronate.

Finally, intravenous pamidronate was well tolerated with minimal and transient side effects, including low-grade fever, myalgia, bone pain and nausea. No patient discontinued treatment because of the toxicity of pamidronate.

In conclusion, these preliminary data suggest that pamidronate represents a safe, well tolerated and effective treatment for the palliation of thyroid cancer patients with symptomatic and osteolytic bone metastases, inducing useful clinical benefits. The induction of the sclerosis of bone lesions observed in a few cases suggests that this treatment may retard disease progression and induce clinical remission of bone metastases. However, the rarity of thyroid cancer with osteolytic bone metastases needs larger, multicentric and, eventually, randomized series, so as to confirm the efficacy of pamidronate therapy in skeletal metastases from thyroid cancer in future trials.

### ACKNOWLEDGEMENTS

We would like to thank Ms Gabriella Granata and Mr Philip Sands for their help in preparation of the manuscript.

### REFERENCES

Aparicio A, Gardner A, Tu Y, Savage A, Berenson J and Lichtenstein A (1998) In vitro cytoreductive effects on multiple myeloma cells induced by bysphosphonates. *Leukemia* **12**: 220–229

Beahrs OH, Henson DE, Hutter RVP and Myers MH (eds) (1988) *Manual for staging of cancer*. 3rd ed. JB Lippincott: Philadelphia

Benford HL, Frith JC, Auriola S, Monkkonen J and Rogers MJ (1999) Farnesol and geranylgeraniol prevent activation of caspases by aminobisphosphonates: biochemical evidence for two distinct pharmacological classes of bisphosphonate drugs. *Mol Pharmacol* **56**: 131–140

Body JJ (1992) Metastatic bone disease: clinical and therapeutic aspects. *Bone* **13**: S57–S62

Cascinu S, Graziano F, Alessandroni P, Ligi M, Del Ferro E, Rossi D, Ficarelli R and Catalano G (1998) Different doses of pamidronate in patients with painful osteolytic bone metastases. *Support Care Cancer*. **6**: 139–143

Cella DF, Tulsky DS, Gray G, Sarafian B, Linn E, Bonomi A, Silberman M, Yellen SB, Winicour P and Brannon J (1993) The functional assessment of cancer therapy scale: development and validation of the general measure. *J Clin Oncol* **11**: 570–579

Coleman RE and Rubens RD (1985) Bone metastases and breast cancer. *Cancer Treat Rev* **12**: 251–270

Coleman RE, Woll PJ, Miles M, Scrivener W and Rubens RD (1988) Treatment of bone metastases from breast cancer with (3-amino-1-hydroxypropylidene)-1, 1 bisphosphonate (APD). *Br J Cancer* **58(5)**: 621–625

*© 2001 Cancer Research Campaign* 

Derenne S, Amiot M, Barille S, Collette M, Robillard N, Berthaud P, Harousseau JL and Bataille R (1999) Zoledronate is a potent inhibitor of myeloma cell growth and secretion of IL-6 and MMP-1 by the tumoral environment. *J Bone Miner Res* **14**: 2048–2056

Gillenwater AM and Weber R (1997) Thyroid carcinoma. *Cancer Treat Res* **90**: 149–169

Grebe SKG and Hay ID (1995) Follicular thyroid cancer. *Endocrinol Metab Clin North Am* **24**: 761–801

Hayward JL, Carbone PP, Heuson JC, Kumaoka S, Segaloff and Rubens RD (1977) Assessment of response to therapy in advanced breast cancer: a project of the programme on clinical oncology of International Union Against Cancer, Geneva, Switzerland. *Cancer* **39**: 1289–1294

Lipton A, Golver D, Harvey H, Grabelsky S, Zelenakas K, Macerata R and Seaman J (1994) Pamidronate in the treatment of bone metastatases: results of 2 dose-ranging trials in patients with breast cancer or prostate cancer. *Ann Oncol* **5(7)**: s31–35

Lupoli G, Cascone E, Arlotta F, Vitale G, Celentano L, Salvatore M and Lombardi G (1996) Treatment of advanced medullary thyroid carcinoma with a combination of recombinant interferon α-2b and octreotide. *Cancer* **78**: 1114–1118

Marcocci C, Pacini F, Elisei R, Schipani E, Ceccarelli C, Miccoli P, Arganini M and Pinchera A (1989) Clinical and biologic behavior of bone metastases from differentiated thyroid carcinoma. *Surgery* **106**: 960–966

Maxon HR III and Smith HS (1990) Radioiodine-131 in the diagnosis and treatment of metastatic well differentiated thyroid cancer. *Endocrinol Metab Clin North Am* **19**: 685–690

Miller AB, Hoogstraten B, Staquet M and Winkler A (1981) Reporting results of cancer treatment. *Cancer* **47**: 207–214

Morton AB, Cantrill JA, Pillai GV, McMahon A, Anderson DC and Howell A (1988) Sclerosis of lytic bone metastases after disodium aminohydroxypropylidene bisphosphonate (APD) in patients with breast carcinoma. *BMJ* **297(6651)**: 772–773

Proye CA, Dromer DH, Carnaille BM, Gontier AJ, Goropoulos A, Carpentier P, Lefebvre J, Decoulx M, Wemeau JL and Fossati P (1992) It is still worthwhile to treat bone metastases from differentiated thyroid carcinoma with radioactive iodine? *World J Surg* **16**: 640–645

Reszka AA, Halasy-Nagy JM, Masarachia PJ and Rodan GA (1999) Bisphosphonates act directly on the osteoclast to induce caspase cleavage of MST 1 kinase during apoptosis. A link between inhibition of the mevalonate pathway and regulation of an apoptosis-promoting kinase. *J Biol Chem* **274**: 34967–34973

Schlumberger MJ (1998) Medical progress: papillary and follicular thyroid carcinoma. *N Engl J Med* **338**: 297–306

Shipman CM, Rogers MJ, Apperley JK, Russel RG and Croucher PI (1997) Bisphosphonates induce apoptosis in human myeloma cell lines: a novel antitumour activity. *Br J Haematol* **99**: 665–672

Tong D, Gillick L and Hendrickson FR (1982) The palliation of symptomatic osseous metastases. *Cancer* **50**: 893–899

Tubiana M, Haddad E, Schlumberger M, Hill C, Rougier P and Sarrazin D (1985) External radiotherapy in thyroid cancers. *Cancer* **55**: 2062–2071

Van Beek E, Pieterman E, Cohen L, Lowik C and Papapoulos C (1999) Nitrogen containing bisphosphonates inhibit isopentenyl pyrophosphate isomerase/farnesyl pyrophosphate synthase with relative potencies corresponding to their antiresorptive potencies in vitro and in vivo. *Biochem Biophys Res Commun* **255(2)**: 491–494

Vitale G, Tagliaferri P, Caraglia M, Rampone E, Ciccarelli A, Bianco AR, Abbruzzese A and Lupoli G (2000) Slow release lanreotide in combination with interferon-α-2b in the treatment of symptomatic advanced medullary thyroid carcinoma. *J Clin Endocrinol Metab* **85**: 983–988

World Health Organization. (1979) WHO Handbook for reporting results of cancer treatment. Geneva

*© 2001 Cancer Research Campaign*
